# Enhancing Pancreatic Beta-Cell Regeneration *In Vivo* with Pioglitazone and Alogliptin

**DOI:** 10.1371/journal.pone.0065777

**Published:** 2013-06-06

**Authors:** Hao Yin, Soo-Young Park, Xiao-Jun Wang, Ryosuke Misawa, Eric J. Grossman, Jing Tao, Rong Zhong, Piotr Witkowski, Graeme I. Bell, Anita S. Chong

**Affiliations:** 1 Department of Surgery, The University of Chicago, Chicago, Illinois, United States of America; 2 Department of Medicine, The University of Chicago, Chicago, Illinois, United States of America; 3 Department of Surgery, Transplant Center, Shanghai Changzheng Hospital, Shanghai, People’s Republic of China; 4 The Institute of Hepatobiliary Surgery, Southwest Hospital, Third Military Medical University, Chongqing, People’s Republic of China; 5 Department of Cellular Transplantation, University of Miami, Coral Gables, Florida, United States of America; 6 Northwestern University Medical Center, Chicago, Illinois, United States of America; 7 The First People’s Hospital of Yunnan Province, Kunming, People’s Republic of China; St. Vincent's Institute, Australia

## Abstract

**Aims/Hypothesis:**

Pancreatic beta-cells retain limited ability to regenerate and proliferate after various physiologic triggers. Identifying therapies that are able to enhance beta-cell regeneration may therefore be useful for the treatment of both type 1 and type 2 diabetes.

**Methods:**

In this study we investigated endogenous and transplanted beta-cell regeneration by serially quantifying changes in bioluminescence from beta-cells from transgenic mice expressing firefly luciferase under the control of the mouse insulin I promoter. We tested the ability of pioglitazone and alogliptin, two drugs developed for the treatment of type 2 diabetes, to enhance beta-cell regeneration, and also defined the effect of the immunosuppression with rapamycin and tacrolimus on transplanted islet beta mass.

**Results:**

Pioglitazone is a stimulator of nuclear receptor peroxisome proliferator-activated receptor gamma while alogliptin is a selective dipeptidyl peptidase IV inhibitor. Pioglitazone alone, or in combination with alogliptin, enhanced endogenous beta-cell regeneration in streptozotocin-treated mice, while alogliptin alone had modest effects. In a model of syngeneic islet transplantation, immunosuppression with rapamycin and tacrolimus induced an early loss of beta-cell mass, while treatment with insulin implants to maintain normoglycemia and pioglitazone plus alogliptin was able to partially promote beta-cell mass recovery.

**Conclusions/Interpretation:**

These data highlight the utility of bioluminescence for serially quantifying functional beta-cell mass in living mice. They also demonstrate the ability of pioglitazone, used either alone or in combination with alogliptin, to enhance regeneration of endogenous islet beta-cells as well as transplanted islets into recipients treated with rapamycin and tacrolimus.

## Introduction

It is now appreciated that insulin-secreting pancreatic beta-cells have a finite life span and that dying beta-cells are continuously replaced throughout life [1]–[6]. Furthermore, insulin-secreting pancreatic beta-cells can further proliferate in response to increasing demand for insulin and after physiological injury [2], [7]–[13]. These observations raise the possibility of enhancing the base-line replication of beta-cells as a therapeutic approach for the treatment of patients with type 1 or type 2 diabetes. Indeed, there is a limited number of clinical case-reports of beta-cell regeneration enabling the complete recovery from type 1 diabetes [14], but in the majority of patients, the reported level of recovery is not sufficient to cure or even maintain glucose homeostasis [12], [13]. In particular there is a concern that regeneration may be greatly reduced or even lost in older individuals [15], [16]. Thus a better understanding of the molecular mechanisms that promote beta-cell proliferation and/or regeneration and the identification of beta-cell replication-based therapies is necessary for beta-cell regeneration to become a clinical reality.

Beta-cell regeneration in mouse models of partial pancreatectomy, autoimmune and streptozotocin (STZ)-induced diabetes have been described using traditional histological assessments [6], [17]–[23]. More recently, two models of endogenous beta-cell regeneration with transgenic mice expressing firefly luciferase under the control of the mouse insulin I promoter (MIP-luc) [24]–[27] or rat insulin promoter [28] have been developed to complement the more traditional models of beta-cell regeneration. These studies demonstrated a tight correlation between beta-cell mass and bioluminescent signal, thereby allowing functional mass beta-cells in living MIP-luc mice to be non-invasively monitored over time. MIP-luc islets can also be transplanted into albino C57BL/6 mice and the functional beta-cell mass of transplanted islets serially monitored [29], [30]. Thus, MIP-luc mice allow for both endogenous and transplanted islet survival and regeneration to be assessed separately, events that may occur simultaneously and contribute to the overall maintenance of normoglycemia. Another important advantage of using the MIP-luc model is that functional islet mass can be serially monitored in individual mice in which normal glycemia is maintained with exogenous insulin, a situation that resembles the diabetic patient [28], [31]–[34].

Here, we investigated the effect of pioglitazone (Pio) and alogliptin on endogenous and transplanted beta-cell regeneration in mice. Pio, a member of the thiazolidinedione drug class, is used in the treatment of patients with type 2 diabetes to increase tissue sensitivity to insulin [35]. Pioglitazone selectively stimulates the nuclear receptor peroxisome proliferator-activated receptor (PPAR)-gamma and to a lesser extent PPAR-alpha, and modulates the transcription of insulin-responsive genes involved in the control of glucose and lipid metabolism in adipose tissues, muscle and liver. Pio has also been shown to preserve insulin secretion and pancreatic morphology in three models of type 2 diabetes [36]–[39]. Consistent with the protective effects of Pio on beta-cell function, Pio has been reported to prevent the development of diabetes induced by multiple low-doses of STZ [40], [41].

Alogliptin is a selective dipeptidyl peptidase IV (DPP-4) inhibitor and a member of a new class of oral medications, which includes sitagliptin, vildagliptin, saxagliptin, linagliptin, for the treatment of type 2 diabetes [42]. DPP-4 inhibitors are designed to slow the inactivation of incretin hormones GLP-1 (glucagon-like peptide-1) and GIP (glucose-dependent insulinotropic peptide). They increase insulin secretion, suppress glucagon secretion and reduce hepatic glucose production. Animal and in vitro studies suggest that DPP-4 inhibitors may also enhance the regeneration and differentiation of pancreatic beta-cells, based in part on the observation that GLP-1 is able to stimulate the proliferation and inhibit apoptosis of beta-cells in vivo and promote the differentiation of beta-cells from human precursor cells (reviewed in [43]). Indeed, long-term treatment with the DPP-4 inhibitor P32/98 (di-[2S,3S]-2-amino-3-methyl-pentanoic-1,3-thiazolidine fumarate) has been reported to preserve and increase beta-cell mass in STZ-induced diabetic rats [44]. However in those studies the effect of the DPP-4 inhibitor was primarily due to protection of beta-cells from STZ toxicity, and its ability to enhance beta-cell regeneration after destruction by STZ was relatively modest.

In this study, we report on the ability of Pio either alone or in combination with alogliptin to enhance endogenous beta-cell regeneration *in vivo*. We also defined the ability of Pio in combination with alogliptin to stimulate an increase in transplanted beta-cell mass in mice treated with rapamycin and tacrolimus and receiving insulin implants to maintain normoglycemia.

## Materials and Methods

### Mice

C57BL/6 or albino C57BL/6 mice (C57BL/6J-*Tyr^c-2J^*) were obtained NCI (Frederick, MD) or Jackson Laboratory (Bar Harbor, ME). MIP-luc transgenic mice (on albino C57BL/6 background) were generated at the University of Chicago, where the transgene comprises the MIP promoter fragment driving the expression of the firefly luciferase (MIP-luc; [25]). Hemizygous MIP-luc transgenic littermates from a single homozygous male MIP-luc mouse crossed with WT albino C57BL/6 females were used in each experiment.

Hemizygous MIP-luc, C57BL/6 albino or wild-type C57BL/6 mice were treated with a single intraperitoneal (IP) injection of STZ (150 mg/kg, Sigma Chemical, St. Louis, MO) to induce diabetes. Mice with non-fasted blood glucose (BG) values >400 mg/dl for more than 2 consecutive days (SureStep; Lifescan, Milpitas, CA) were considered diabetic. Hyperglycemia was controlled by the sub-cutaneous implantation of a single LinBit insulin tablet (Linshin, Canada Inc). All studies were performed in accordance to protocols approved by the University of Chicago Institutional Animal Care and Use Committee.

### Islet Isolation and Transplantation

Syngeneic islets from MIP-luc transgenic C57BL/6 mice were isolated following intraductal collagenase digestion (Collagenase P, 0.3 mg/ml; Roche, Indianapolis, IN) and purification by Ficoll gradient centrifugation (Sigma, St. Louis, MO) as previously described [17]. Approximately 200 C57BL/6 or 50-75 MIP-luc islets were transplanted under the kidney capsule as previously described [17].

### Bioluminescent imaging

Bioluminescent optical imaging was performed using a IVIS Spectrum (Caliper Life Science Inc, Hopkinton, MA) imaging system. Briefly, MIP-luc mice were fasted for 4 h, shaved, then anesthetized with isofluorane as previously described [6]. Mice were placed on their sides on the imaging stage and an overlay image was initially taken. Mice were then injected IP with 15 mg/ml D-luciferin in sterile PBS (150 mg/kg) and after exactly 14 min, a bioluminescent image was captured utilizing an exposure time of 1 minute. Subsequent image processing including quantification of bioluminescence was conducted using Living Image Software v. 4.2 (Caliper).

### Drug Administration

Alogliptin and Pio was suspended in 0.5% carboxy-methyl-cellulose (CMC) and a daily dosage of 15 mg/kg/day or 45 mg/kg/day of alogliptin (Takeda) or 25 mg/kg Pio (Takeda) was administered 5 days a week for the indicated duration. Sirolimus (Wyeth) was administered as a loading dose of 0.2mg/kg on day 1, followed by 0.1 mg/kg for the duration of the study [45]. Tacrolimus (Astellas) was dosed at 1 mg/kg [45]. All drugs were delivered orally in a total volume of 100 µl by gastric tube, 5 days per week as indicated.

### Intraperitoneal Glucose Tolerance Test (IPGTT)

At the end of some experiments, an intraperitoneal glucose tolerance test (IPGTT) was performed as previously described [17]. After four hours of fasting, mice received an intraperitoneal injection of dextrose (2 g/kg) and BG levels determined from the tail vein at 30-minute intervals. Two days after the IPGTT test, the mice were sacrificed and histology performed to quantify beta-cell mass.

### Histological Studies

Beta-mass was determined as described previously [17]. Briefly, the pancreas was removed, weighed, fixed, embedded and each block was serially sectioned (5 µm thickness) at 100-μ m intervals and sections were stained with anti-porcine insulin polyclonal antibody (Dako, Carpenteria, CA) and biotinylated secondary antibody (HistoMouse-Plus, Zymed, South San Francisco, CA). Quantitative evaluation was performed with a Nikon Eclipse E800M microscope (Nikon Instruments, Melville, N.Y.) with an Olympus Q-Color3 video camera (Optical Analysis Corp., Nashua, N.H.). The area of insulin-positive cells and total pancreatic area were evaluated in each stained section (five sections per mouse). Overall beta cell–relative volume in each mouse was determined as the mean of the ratio of the area occupied by insulin-positive cells to the area occupied by total pancreatic cells in each section multiplied by section thickness. Total beta-cell mass per pancreas was calculated by multiplying the total pancreatic weight and relative beta-cell volume.

### Statistics

Data are presented as means ± SEM and evaluated for statistical significance by ANOVA (Prism). A value of *p*<0.05 was considered to be statistically significant.

## Results

### Islet Beta-cell Regeneration: Effect of Pioglitazone

**Figure 1:**
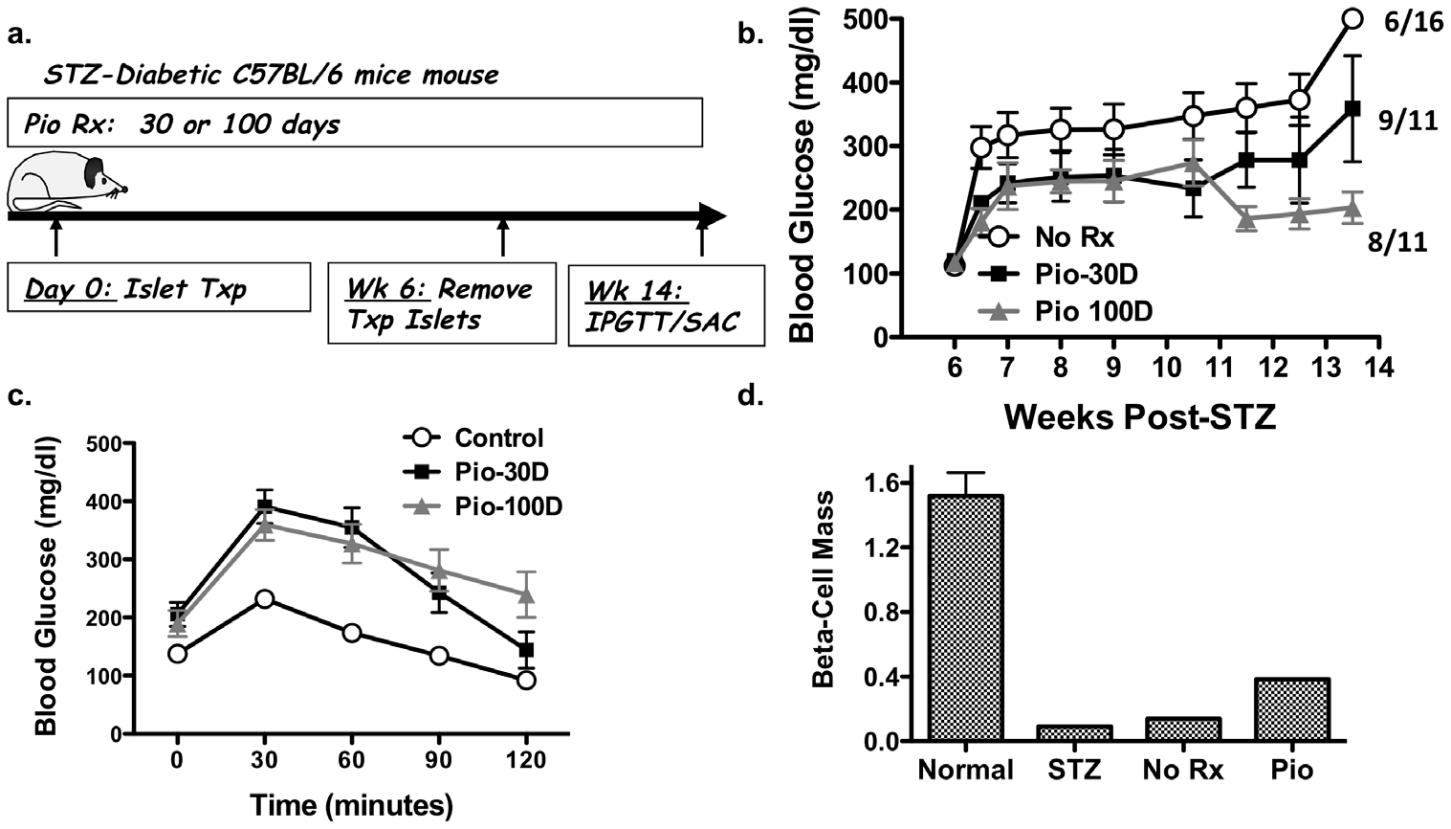
Effect of pioglitazone on endogenous beta-cell regeneration. (a) Overview of experimental design. Following treatment with STZ, diabetic mice (BG >400 mg/dl) received approximately 200 syngeneic C57BL/6 islets under the kidney capsule for 6 weeks (Islet Txp), and then underwent a nephrectomy to remove the transplanted islets. (b) BG was monitored every 1–2 weeks after nephrectomy and the number of mice with normoglycemia (<250 mg/dl) at ≥13 weeks post-STZ ± Pio treatment for 30 or 100 days (Pio-30D or Pio-100D) are indicated. Data are presented as mean (N = 11–16/group) ± standard error of mean (SEM). (c) After 4 h fasting, IPGTT was performed on only the mice with normoglycemia (<250 mg/dl) at ≥12 weeks post-STZ compared to control non-diabetic C57BL/6 mice. (d) Pancreatic beta-cell mass at week 2 post-STZ or week 14 post-STZ and islet transplant receiving CMC (No Rx) or Pio (25 mg/kg/day; Pio) of 3–4 randomly selected mice still alive and with normoglycemia (<250 mg/dl). Data are mean beta-cell mass of 3–4/group (mg/pancreas ± SEM).

We first investigated the effect of Pio in a previously reported model of functional beta-cell regeneration in the native pancreas following STZ-induced diabetes [17]. Diabetic C57BL/6 mice received ≥200 syngeneic islets under the kidney capsule to maintain normoglycemia for 6 weeks (mean BG ≤150 mg/dl) (Fig. 1a). The transplanted islets were then removed and BG monitored for a further 8 weeks. Functional regeneration of the islet beta-cells in the pancreas, defined as the maintenance of random BG levels at <250 mg/dl after the removal of the transplanted islets, was observed in 37.5% (6 of 16) of the untreated mice, with a mean BG of 340±36 mg/dl over the 8 week monitoring period. In mice that received 25 mg/kg/day Pio for 30 days from the onset of STZ-induced diabetes, stable normoglycemia (<250 mg/dl) was observed in 9 of the 11 mice (Fig. 1b). The mean BG levels in the 30-day Pio-treated mice over the monitoring period was 242±85 mg/dl and was significantly lower (p≤0.01) than the untreated group. To test whether the effect of Pio could be improved, we extended treatment from 30 to 100 days. Stable normoglycemia was observed in 8 of the 11 mice and the overall BG level of the 100-day Pio-treated mice was 219±22 mg/dl. Thus, treatment for 100 days with Pio resulted in significantly lower (p≤0.005) BG levels compared to the untreated and 30-day Pio-treatment groups.

IPGTT was performed on mice treated with Pio (30 or 100 day) and with BG levels of ≤250 mg/dl (Fig. 1c). BG levels at 120 min post-glucose challenge returned to baseline in 3 of 5 (60%) mice treated with Pio for 30 days, and 6 of 8 (75%) mice treated with Pio for 100 days. Thus Pio treatment for 30 days or continuously for 100 days was able to partially restore glucose tolerance, compared to the IPGGT response in non-diabetic mice. We also quantified beta-cell mass by histology in 3–4 randomly selected mice with BG of <250 mg/dl from the 100-day Pio treatment group (Fig. 1d) and observed that Pio treatment resulted in a modest 4-fold enhancement in beta-cell mass compared to acute (day 14–20) post-STZ group, and a 2.7-fold increase over the non-Pio treated group (day 100 post-STZ). While these data suggest the ability of Pio to promote beta-cell regeneration, the modest increase in beta-cell mass and the inability to determine the kinetics of beta-cell regeneration prompted further investigation into the regenerative effects of Pio in MIP-luc mice.

### Effect of Pioglitazone on Endogenous Beta-cell Regeneration in STZ-induced Diabetic MIP-luc Mice

**Figure 2:**
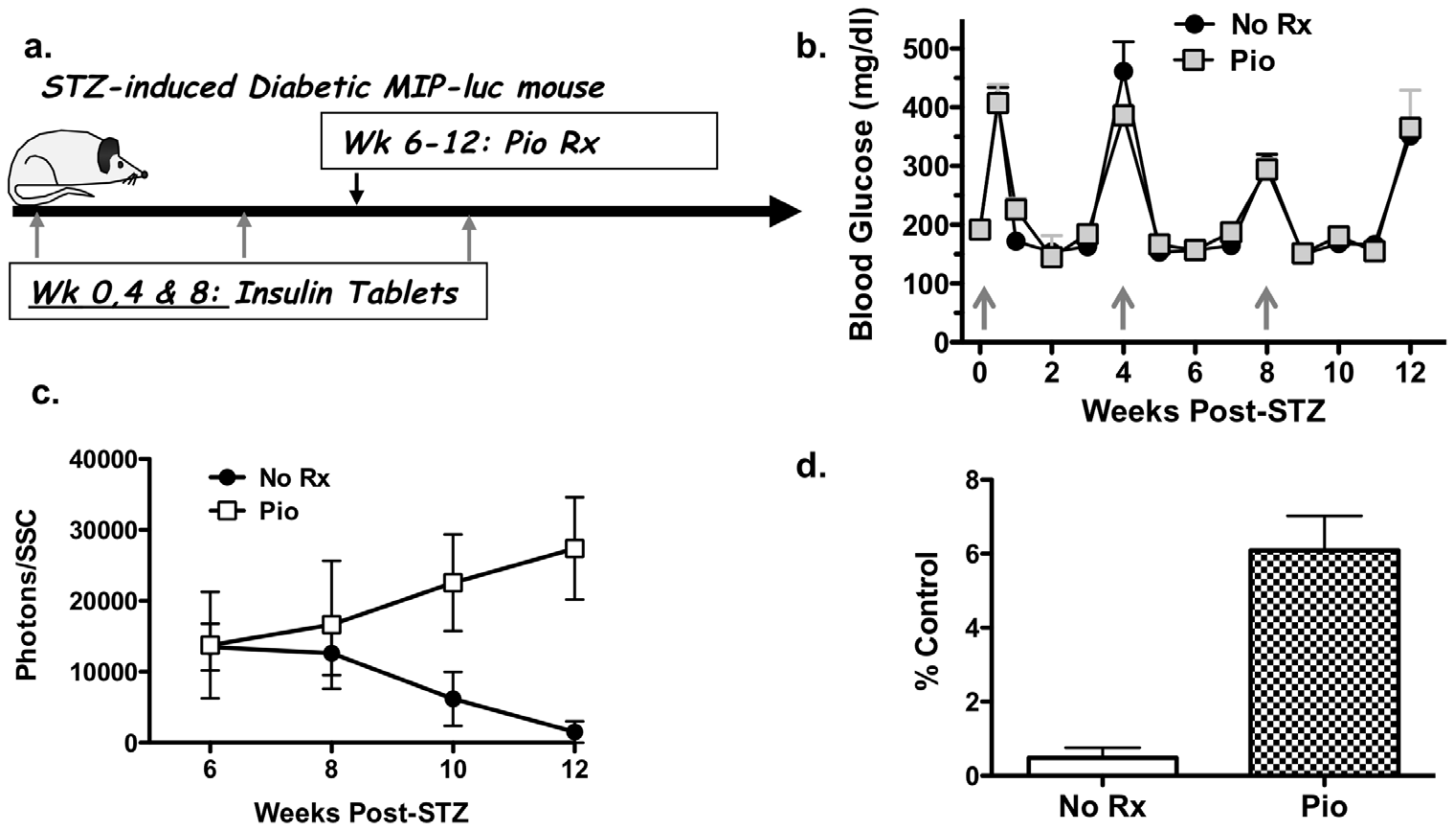
Effect of pioglitazone on endogenous beta-cell regeneration in MIP-luc mice. (a) Overview of experimental design. Following treatment with STZ, diabetic MIP-luc mice (BG >400 mg/dl) received an insulin implant at week 0, 4 and 8 post-STZ (grey arrows). Mice were divided into two groups at 6 weeks post-STZ, receiving CMC (No Rx) or Pio (25 mg/kg; 5X/week; Pio). BG (b) and the bioluminescent signal from the endogenous beta-cells (c) were monitored every week. Data are presented as mean (N = 5/group) ± standard error of mean (SEM). (d) Bioluminescent signal at week 12 post-STZ in control (No Rx) or Pio-treated groups as a mean percentage of bioluminescence prior to STZ treatment.

We previously reported that while STZ-treatment acutely induced diabetes, the beta-cell mass continued to decline for up to 4–6 weeks post-STZ treatment [6]. Thus to test the efficacy of Pio in promoting the regeneration of endogenous islet beta-cells, we initiated treatment at 6 weeks post-STZ treatment (Fig. 2a). STZ-treated albino C57BL/6 MIP-luc mice, with non-fasted BG values >400 mg/dl for 2 consecutive days, received insulin implants to control BG for up to 4 weeks, whereupon the BG levels rose to >250 mg/dl and another insulin tablet was implanted (Fig. 2b). Prior to initiation of treatment, the bioluminescent signal in each mouse was determined and observed to be approximately 3% of the signal prior to STZ-treatment, and the mice were divided into two groups (N = 5/group) with equivalent mean bioluminescent intensities. Treatment with 25 mg/kg Pio resulted in a gradual increase in the bioluminescent signal, culminating in a 12.6 fold higher bioluminescent signal compared to the untreated control at 12 weeks post-STZ treatment (Fig. 2c; p<0.01). The final bioluminescent signal in the Pio-treated group at 12 weeks post-STZ was 6.1±2.7% of initial signal before STZ treatment, compared to 0.4±0.6% in the untreated group (Fig. 2d). Thus consistent with our observations described in Fig. 1, Pio-treatment starting at 6 weeks after STZ-induced diabetes, when the majority of beta-cells were destroyed, was able to significantly enhance bioluminescent signal (p<0.01), nonetheless, there remained insufficient endogenous beta-cells to maintain normoglycemia after 6 weeks treatment with Pio (Fig. 2b).

### Effect of Pioglitazone in Combination with Alogliptin on Beta-cell Regeneration in STZ-induced Diabetic MIP-luc Mice

**Figure 3:**
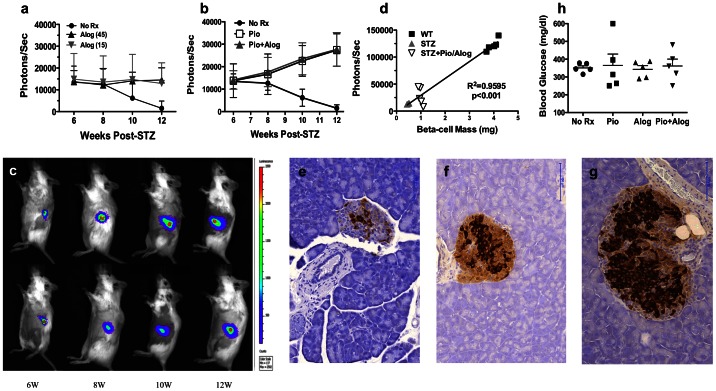
Effect of alogliptin alone or in combination with pioglitazone on endogenous beta-cell regeneration. Bioluminescent signal (Photons/sec) from endogenous pancreas of albino MIP-luc mice treated with (a) aloglipin alone (Alog 15 or 45 mg/kg/day; 5X/week) or (b) alogliptin (45 mg/kg/day; 5X/week) plus Pio (Pio+Alog; 25 mg/kg/day; 5X/week) from week 6–12 post-STZ. The control No Rx group in (a) and (b) is from Fig. 2a. (c) Representative bioluminescent images of mice treated with Pio+Alog (top) and Pio (bottom) for 6, 8, 10 and 12 weeks. Overall experimental design was as described for Fig. 2. (d) Correlation between bioluminescent signal and histological measure of beta cell mass are provided for the STZ only, STZ with Pio plus alogliptin (Pio/Alog) and untreated, non-diabetic WT mice (WT) (N = 5/group). Representative histology (20X magnification; bar = 100 µm) and correlation between bioluminescent signal and histological measure of beta-cell mass from STZ-induced diabetic mice that were maintained with insulin implants and were untreated (e) or treated from week 6–12 post-STZ with alogliptin plus Pio (f), and sacrificed at 12 weeks post-STZ. Islets from untreated non-diabetic mice are illustrated (γ). Random BG levels at 12 weeks post-STZ (4 weeks after last insulin implant) for the untreated (No Rx), alogliptin only (Alog; 45 mg/kg/day), Pio only (25 mg/kg/day) and both (Pio+Alog) are illustrated in (h). Data are presented as means ± SEM.

Based on a recent report that alogliptin added to Pio improved glycaemic control in patients with type 2 diabletes [46], we tested whether alogliptin could improve on the effects of Pio in STZ-induced diabetic MIP-luc mice. At the initiation of treatment with alogliptin alone or in combination with Pio, the bioluminescent intensities in all the groups (N = 5/group) were equivalent. In mice receiving alogliptin alone, either at the 15 or 45 mg/kg dose, the beta-cell mass remained constant over the 6-week treatment group while the beta-cell mass in the control group continued to decline (Fig. 3a). However the differences between the alogliptin-treated and untreated groups were not significant.

**Figure 4:**
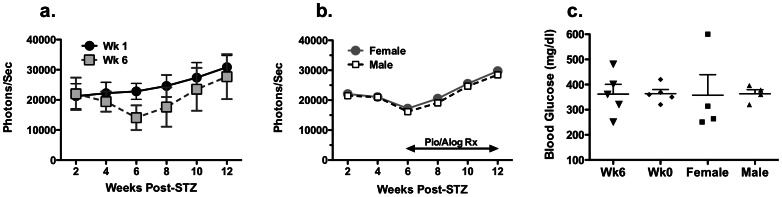
Effect of early treatment with pioglitazone plus alogliptin, or of gender on endogenous beta-cell regeneration. Bioluminescent signal in mice treated with (a) Pio (25 mg/kg/day; 5X/week) plus alogliptin (45 mg/kg/day; 5X/week) starting from week 1–12 (Wk 1) or from week 6–12 (Wk 6) post-STZ treatment. Bioluminescent signal in (b) male or female mice treated with Pio (25 mg/kg/day; 5X/week) plus alogliptin (45 mg/kg/day; 5X/week) starting from week 6–12 post-STZ treatment. (c) Random BG levels at 12 weeks post-STZ. Data are presented as means ± SEM (N = 4–5/group).

We also investigated whether Pio in combination with alogliptin would result in further enhancement of beta-cell regeneration (Fig. 3b and c). While a significant increase in the bioluminescent signal was observed at 6 weeks following treatment with Pio in combination with alogliptin (p<0.01) compared to the untreated group, this increase was not significantly better than Pio alone. We also confirmed that the bioluminescent signal in this group receiving Pio plus alogliptin correlated with modestly increased beta-cell mass with standard histological assessment (Fig. 3d-g). Finally, consistent with observations with the Pio only treatment group, mice treated with Pio plus alogliptin for 6 weeks remained hyperglycemic after insulin treatment was stopped underscoring the modest regenerative capacity of beta-cells in the MIP-luc mice (Fig. 3h). We also tested whether extending the treatment period with Pio plus alogliptin would enhance beta-cell regeneration by initiating treatment from the day of diabetes diagnosis instead of waiting for 6 weeks afer STZ-treatment. This early treatment with Pio plus alogliptin prevented the early decline in the beta-cell mass observed in mice that did not receive Pio plus alogliptin till week 6 post-STZ (Fig. 4a). Disappointingly, both groups had comparable bioluminescence and random BG levels at 12 weeks post-STZ-treatment (Fig. 4a and c).

One of the most robust physiological stimuli of beta-cell expansion is pregnancy [47], however whether non-pregnant females have superior capacity over males to undergo beta-cell regeneration following their destruction with STZ is currently unclear. We tested whether gender affected the rate of beta-cell regeneration by comparing age-matched male and female mice treated with Pio plus alogliptin. As shown in Fig. 4 (panels b and c), both STZ-induced diabetic males and females had comparable rates of restoration of bioluminescent signal and BG levels in response to Pio.

### Transplanted Islet Beta-cell Mass: Effect of Alogliptin in Combination with Pioglitazone

**Figure 5:**
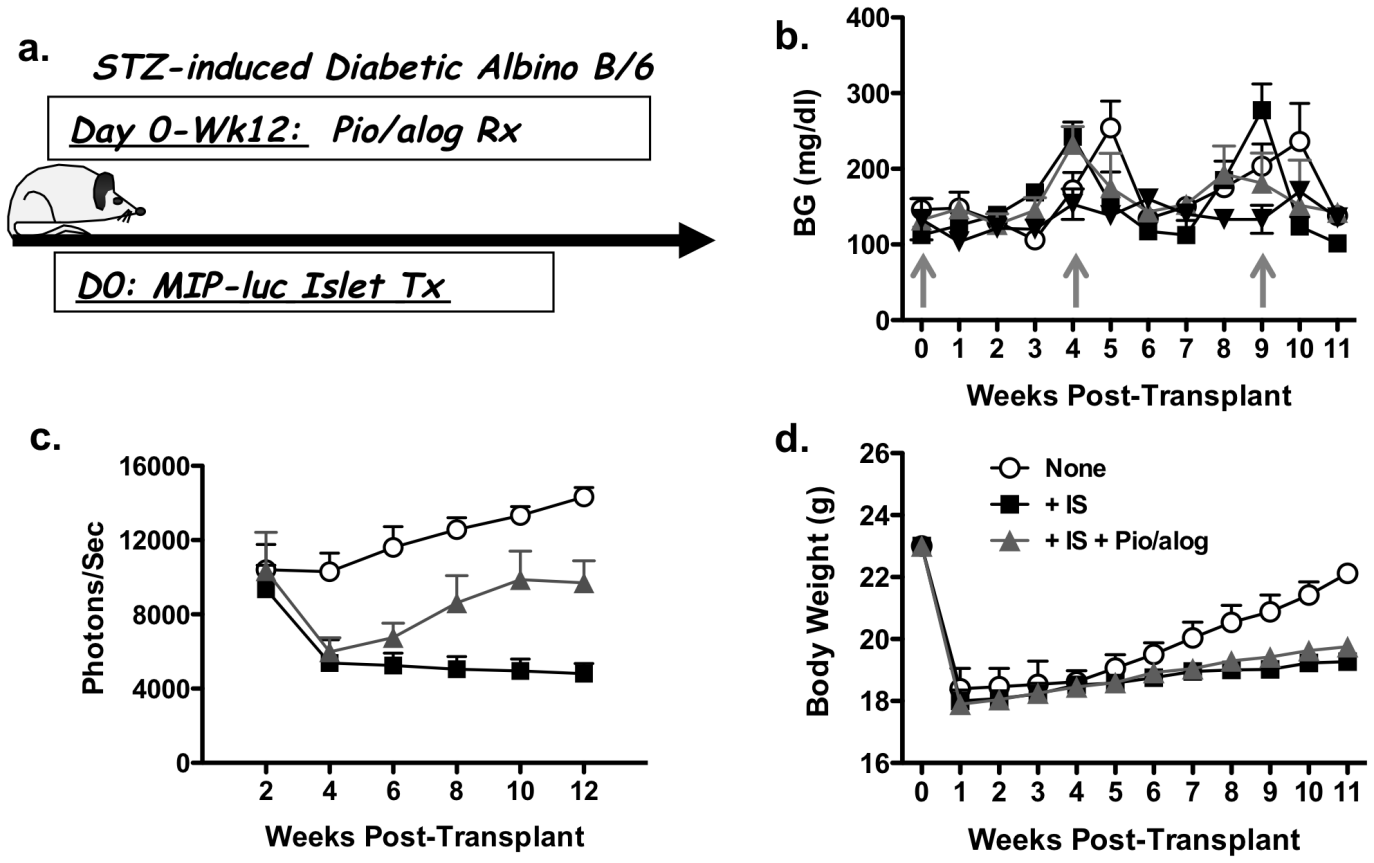
Effect of immunosuppressive drugs and pioglitazone and alogliptin on syngeneic transplanted beta-cell regeneration. (a) Overview of experimental design. Following treatment with STZ, diabetic albino C57BL/6 mice received 50–75 MIP-luc syngeneic islets transplanted under the kidney capsule and insulin implants at week 0, 4–5 and 9–10 post-STZ, when BG levels were >350 mg/dl (grey arrows in (b)). Mice were divided into three groups following islet transplantation: no immunosuppression (None), receiving immunosuppression (IS) of rapamycin (0.2 mg/kg on day 1, followed by 0.1 mg/kg for the duration of the study) and tacrolimus (1 mg/kg) (+ IS), and IS+Pio (25 mg/kg; 5X/week) plus alogliptin (+ IS+Pio/alog 45 mg/kg/day; 5X/week). Weekly random BG (right) levels (b), bioluminescent intensity every two weeks (c) and weekly body weights (d) were determined. Data are presented as means ± SEM (N = 5/group).

Recent reports from clinical islet transplantation indicate improvement in the outcomes of islet transplantation, although approximately 44% of recipients become insulin dependent at 3 years after successful islet transplantation [48]–[50]. Possible reasons for this return to insulin dependence include islet-toxicity associated with the Edmonton immunosuppression (IS) protocol of rapamycin plus tacrolimus [2], [51] and metabolic exhaustion as a result of sub-optimal beta-cell mass [52]. To define the combined effects of sub-optimal islet mass, immunosuppression, Pio and alogliptin, we developed a model of syngeneic islet transplantation where only 50–75 MIP-luc islets were transplanted under the kidney capsule of syngeneic albino C57BL/6 mice that had been pre-treated with STZ to induce diabetes (Fig. 5a). These mice also received insulin implants every 4–5 weeks to maintain normoglycemia (Fig. 5b), and bioluminescence of the transplanted syngeneic islets was determined every 2 weeks (Fig. 5c).

At 1 week post-STZ, the transplanted mice were divided into three groups to ensure comparable bioluminescence: Control (No IS); sirolimus plus tacrolimus (IS), and IS+Pio plus alogliptin, starting from week 1- 12 (IS+Pio/alog) (N = 5/group). In the absence of immunosuppression, the bioluminescent signal from the transplanted islets gradually increased by an average of 37.6% over the 10-week post-transplant period. In the IS group, the bioluminescent signal rapidly dropped by 42.5% from week 2–4 (p<0.05) and then stabilized from week 6–12. In the IS group receiving Pio plus alogliptin, the bioluminescent signal also dropped by 42% from week 2–4, but this was followed by an increase in beta-cell bioluminescence from week 6–12 so that by week 12 post-STZ treatment, the bioluminescent signal was increased by 165% from the nadir at week 4 (p<0.05; Fig. 5c). The significantly improved bioluminescent signal in mice receiving IS in combination with Pio plus alogliptin over those receiving IS alone was not associated with differences in body weight, which were comparable in both groups (Fig. 5d). Thus treatment with Pio plus alogliptin was able to overcome immunosuppression-induced toxicity and modestly increase the mass/function of transplanted beta-cells.

## Discussion

Recent reports of functional pancreatic beta-cell regeneration in murine models [18], [53] raise the exciting possibility that beta-cell regeneration may be a treatment for patients with autoimmune as well as non-autoimmune diabetes [54]–[57]. Despite experimental evidence that beta-cells can regenerate from residual beta-cells or beta-cell precursors [18], [53] [2], [19], much remains unresolved. Some of the current challenges include the need to further understand the processes controlling islet beta-cell regeneration, identify therapeutic agents that significantly enhance regeneration in rodent models, and translate these insights into viable approaches for enhancing beta-cell regeneration and curing diabetes in the clinic.

Our previous reports suggest that beta-cell regeneration in STZ-diabetic mice can occur when normoglycemia is maintained by insulin implants or islet transplantation [6], [17]. However regeneration is slow and modest, and the methods for quantifying regeneration are time consuming and require sacrifice of the experimental mouse. Thus, there is an urgent need to develop better experimental models that allow for the serial quantification of beta-cell regeneration in living mice, and to identify reagents that enhance endogenous beta-cell regeneration. Here we used bioluminescent signals from MIP-luc mice to quantify changes in functional beta-cell mass in live mice. To control for possible insertional effects of the MIP-luc transgene, hemizygous MIP-luc transgenic littermates from a single homozygous male MIP-luc mouse crossed with WT albino C57BL/6 females were used in each experiment. In our model, the regenerating beta-cells are protected from hyperglycemia either with transplanted islets or insulin implants; the latter situation more closely resembling the treatment of diabetic patients. Despite the advantages to this model and the previous demonstration that that beta-cell mass at steady state is correlated with the bioluminescent signal intensity [6], [24]–[27], [29], [30], we acknowledge a major caveat of the MIP-luc model is that increased bioluminescent signal could reflect increased transcription of the insulin I gene promoter or increased ATP in the beta-cells in the absence of increased beta-cell mass. Thus, we consider the increased bioluminescent to be a *in vivo* measure of functional beta-cell mass and immunohistological staining at the end of the experiment to confirm that changes in bioluminescence coincide with comparable changes in beta-cell mass.

A number of reagents that have been reported to preserve beta-cell function and mass in rodents, including exendin-4, EGF, gastrin and lisofylline [58] [59], [60] [61], [62]. Because the GLP-1 mimetic, exendin-4 is effective at preservinγ beta-cell mass in multiple models of beta-cell dysfunction and destruction [60], [63]–[65], we predicted that DPP-4 inhibitors that potentiate the half-life of GLP-1 would also facilitate beta-cell regeneration [66]. However, in our studies, the DPP-4 inhibitor, alogliptin, did not significantly enhance endogenous beta-cell regeneration, nor did it enhance the effects of Pio.

Pio has previously been shown to prevent islet cell apoptosis and the development of diabetes induced by multiple low-doses of STZ [40] and in db/db mouse models of progressive beta-cell dysfunction and destruction [67], [68]. Using our model of STZ-diabetic MIP-luc mice, treatment of Pio plus alogliptin starting from the time of diabetes diagnosis when the bioluminescent signal was 0.5–8% of baseline prior to STZ-treatment, prevented the further decay in bioluminescent signal observed in non-Pio-treated controls. However when Pio treatment is initiated at the time of diagnosis of diabetes, it is unclear whether the effects of Pio are due to its ability to prevent beta-cell death, or whether Pio has additional effects on beta-cell regeneration. To address the latter possibility, Pio treatment was delayed for 6 weeks to allow the STZ toxicity to resolve. Delayed Pio plus alogliptin treatment remained able to enhance the bioluminescent signal, confirming the ability of Pio to not only preserve but also increase beta-cell mass; a finding that was confirmed by histology.

The observation that patients successfully transplanted with pancreatic islets return to insulin-dependence after 5 years has been explained by drug toxicity, metabolic stress or inadequate immunosuppression and point to a need to enhance the function of transplanted beta-cells. We developed a model of transplantation of sub-optimal beta-cell mass under the kidney capsule of STZ-diabetic albino C57BL/6 recipients, using transplanted islets from syngeneic MIP-luc mice. This model eliminated concerns of inadequately controlled alloreactivity and beta-cell toxicity as a result of unconstrained BG levels, and allowed a focus on the effects of suboptimal beta-cell mass and toxicity of the immunosuppression regimen. Contrary to our expectation that immunosuppression toxicity would be cumulative and the loss of islet mass would be incremental over time, we observed that the combination of rapamycin and tacrolimus caused a rapid decline in bioluminescent signal at 2–4 weeks post-transplantation, after which the bioluminescent signals stabilized despite continued rapamycin and tacrolimus treatment. These observations suggest that immunosuppression itself may not be toxic, but together with inflammation in the early post-transplant period are the primary cause of beta-cell destruction. We further demonstrate that Pio plus alogliptin treatment, initiated from the day of transplantation, could not prevent the early decline in bioluminescent signal but was able to significantly increase the bioluminescent signal from day 28–90. These experiments provide, for the first time, insight into the kinetics of beta-cell destruction by immunosuppression in recipients with sub-optimal beta-cell mass but who maintained normoglycemia with insulin implants. They also demonstrate the ability of Pio plus alogliptin to enhance the functional mass of the transplanted beta cells and identify the kinetics of their therapeutic effects, and delineate a critical period for additional intervention in the early post-transplantation period to preserve beta-cell mass and in the later period to further promote beta-regeneration.

In summary, we have used the MIP-luc mouse model to study the fate of both endogenous as well as transplanted islets in diabetic mice surviving on exogenous insulin, using beta-cell bioluminescence as a surrogate measure of function and mass. We also report on the efficacy of Pio, alone or in combination with alogliptin, in enhancing beta-cell regeneration. However, promotion of beta-cell regeneration by Pio is relatively modest, and much work is additionally required to define the basis for the increased beta-cell bioluminescence by Pio, and to test whether Pio in combination with other agents may further promote endogenous as well as transplanted beta-cell regeneration.
